# Surgery or radiotherapy for early-stage cancer study protocol for an emulated target trial of radical radiotherapy versus radical cystectomy, with either following neoadjuvant chemotherapy, for organ-confined muscle-invasive bladder cancer

**DOI:** 10.1136/bmjopen-2026-119596

**Published:** 2026-06-11

**Authors:** Eva Kagenaar, David Gibran Lugo-Palacios, Hannah Bromley, Ajay Aggarwal, Andrew Hutchings, Stephen O’Neill, Bernard Rachet, Jo Cresswell, Ananya Choudhury, Richard Grieve

**Affiliations:** 1 Department of Health Services Research and Policy, London School of Hygiene and Tropical Medicine Faculty of Public Health and Policy, London, UK; 2 Department of Clinical Oncology, The Christie National Health Service (NHS) Foundation Trust, Manchester, UK; 3 South Tees Hospitals NHS Trust, Middlesbrough, UK; 4 Division of Cancer Sciences, The University of Manchester, Manchester, UK

**Keywords:** Adult oncology, RADIOTHERAPY, Adult surgery

## Abstract

**Abstract:**

**Introduction:**

Neoadjuvant chemotherapy (NAC) followed by definitive treatment consisting of either radical radiotherapy or radical cystectomy is the recommended treatment for patients with organ-confined muscle-invasive bladder cancer (OC-MIBC). A randomised controlled trial (RCT) aimed to compare the effectiveness of radical radiotherapy and radical cystectomy but failed to recruit. Radical radiotherapy is non-invasive and organ-preserving, and observational studies have suggested this treatment may be associated with similar outcomes compared with radical cystectomy. However, in these observational studies, the risk of confounding was high, and they did not consider the receipt of NAC. The surgery or radiotherapy (SORT) for the early-stage cancer study will assess the comparative effectiveness and cost-effectiveness of either radical cystectomy or radical radiotherapy, both after NAC for OC-MIBC. We will use a target trial emulation approach to reduce the risk of bias when assessing comparative effectiveness from observational data.

**Methods and analysis:**

The SORT study will use UK’s National Cancer Registry to identify individuals diagnosed with urothelial OC-MIBC (T2-4aN0M0) between 1 January 2015 and 31 December 2021 who received either radical radiotherapy or radical cystectomy after NAC. The data will be linked to Hospital Episode Statistics (HES), National Radiotherapy Data Set (RTDS) and Systemic Anti-Cancer Therapy (SACT) data sets to gather information on clinical, tumour and socio-demographic characteristics and receipt of treatment. Using the target trial emulation framework, we will define the eligibility criteria and radical radiotherapy and radical cystectomy receipt. To reduce the risk of confounding, we will use advanced statistical approaches to allow for differences in measured baseline characteristics between the comparison groups.

The primary outcome is 3-year all-cause mortality after radical treatment receipt. Secondary outcomes will include all-cause and bladder-cancer-associated mortality at 3 and 5 years, time to death, incremental costs and incremental cost-effectiveness reported according to net health benefits.

**Ethics and dissemination:**

The study was approved by the London School of Hygiene and Tropical Medicine Ethics Committee (Reference number 29717 - 1). Results will be communicated in open-access journals and conferences to clinicians, researchers, patients and policymakers.

**STRENGTHS AND LIMITATIONS OF THIS STUDY**

- The surgery or radiotherapy study will use England’s Cancer Registry to include a nationally representative sample and provide evidence of the comparative effectiveness of radical radiotherapy versus radical cystectomy, both following neoadjuvant chemotherapy (NAC) for the entire population of interest.
- The risk for confounding and biases will be reduced by combining the emulated target trial framework with statistical methods that reduce the risk of bias from differences in observed baseline characteristics between the comparison groups.
- One limitation is that there may be unobserved baseline differences between the comparison groups, for example, about staging after NAC, especially for patients who have radical radiotherapy.

## Introduction

 From 2017 to 2019, there were approximately 5600 bladder cancer deaths in the UK, making it the ninth most common cause of cancer deaths in the UK.^1^ In England, survival outcomes following bladder cancer irrespective of the stage of diagnosis are worse than in comparable countries.^2^ While the majority of patients diagnosed have bladder cancer that has not penetrated the muscle wall in the bladder (non-muscle invasive bladder cancer), around 20% are diagnosed with a cancer that has spread to the muscle wall of the bladder but not yet to nearby or distant lymph nodes or sites, referred to as organ-confined muscle-invasive bladder cancer (OC-MIBC).^3^

Individuals with OC-MIBC should be considered for treatment with curative intent. The National Institute for Health Care and Excellence (NICE) recommends neoadjuvant chemotherapy (NAC) with a cisplatin combination regimen followed by definitive treatment, comprising radical cystectomy or radical radiotherapy, for individuals newly diagnosed with OC-MIBC.^4^ Radical cystectomy can be required for individuals who require a urinary stoma or continent urinary diversion (bladder substitution or a catheterisable reservoir).^4^ Radical radiotherapy can be offered as an organ-preserving, non-invasive treatment for patients who are unfit for surgery or who do not want to undergo surgery.^4^ However, for most patients, radical cystectomy or radical radiotherapy are curative-intent treatment options.^4^ Ultimately, the choice of definitive treatment should be made in joint consultation with the patient and multidisciplinary clinical team.^4^ In the UK, there is wide variation in treatment receipt for OC-MIBC. Despite NICE recommendations, less than half of individuals who have radical treatment for OC-MIBC receive NAC.^5 6^ Furthermore, wide variation in the proportion of individuals who receive radical radiotherapy as definitive treatment for MIBC has been observed across geographical regions^7 8^ and socio-demographic subgroups.^5 9^

There is clinical equipoise about the optimal management of OC-MIBC. While RCTs could provide the required evidence on comparative effectiveness to reduce clinical uncertainty, the SPARE trial that aimed to compare radical cystectomy or radical radiotherapy, both after NAC, failed to recruit due to patient and clinician preference.^10^ Several observational studies have compared radical cystectomy and radical radiotherapy, but the results are unclear. The majority of observational studies report that overall survival is similar for patients undergoing radical cystectomy versus radical radiotherapy.^11^,^13^ However, some of these studies report improved overall survival among patients who receive radical radiotherapy,^14^ others among those who receive radical cystectomy.^15^ The majority of these observational studies include patients who did not receive NAC to increase sample size, despite available evidence that NAC improves survival.^11^,^1416^ Three meta-analyses have pooled the results of observational studies.^16^,^18^ Two of these studies reported that overall survival is similar for radical cystectomy versus radical radiotherapy.^16 18^ However, a third meta-analysis reported that all-cause survival was better for patients who had radical cystectomy after being downstaged following NAC.^17^ The meta-analyses have problems with their design, in that they all have relatively small sample sizes and important differences across the designs of the observational studies that were included.^16^,^18^

There is limited and inconclusive evidence about the relative cost-effectiveness of radical radiotherapy versus radical cystectomy as definite treatments after NAC for early-stage OC-MBIC.^19^,^23^ Two cost-effectiveness analyses (CEA) reported that radical radiotherapy was more cost-effective.^21 23^ A third CEA found that radical cystectomy was more cost-effective for patients aged 65 and over. ^23 24^All of these CEAs were conducted in North America,^21–23^which makes it difficult to apply the results to wider contexts.

The lack of high-quality, consistent evidence about the comparative clinical effectiveness and cost-effectiveness of radical radiotherapy versus radical cystectomy contributes to clinical uncertainty about the choice of definitive treatment, and there are wide variations in utilisation across the National Health Service (NHS) in the UK. Improvements in the quality and availability of routinely collected data offer the opportunity to address common limitations in the assessment of comparative effectiveness and cost-effectiveness from observational data. In particular, the target trial emulation framework can reduce the risk of confounding by conceptualising a non-randomised study as if it were an RCT.^25^ Hence, this study will leverage the variation across NHS trusts and over time in routine data in the uptake of radical radiotherapy versus cystectomy after NAC for OC-MIBC. We will use a target trial emulation design combined with advanced statistical methods to reduce the inevitable risk of confounding when using these routine data to assess comparative effectiveness and cost-effectiveness.

## Aims and objectives

The surgery or radiotherapy (SORT) study aims to assess the comparative effectiveness and cost-effectiveness of NAC followed by radical radiotherapy versus NAC followed by radical cystectomy for urothelial OC-MIBC (T2-T4aN0M0) using the English Cancer Registry data from the National Disease Registration Service (NDRS).^26^

The study objectives are:

1. To assess the comparative effectiveness of NAC followed by radical radiotherapy vs NAC followed by radical cystectomy for urothelial OC-MIBC (T2-T4aN0M0) for the overall population and across subgroups;
2. To evaluate the comparative cost-effectiveness of NAC followed by radical radiotherapy or NAC followed by radical cystectomy for urothelial OC-MIBC (T2-T4aN0M0) for the overall population and across subgroups;

## Methods and analysis

### Overview

The SORT study will use nationally representative linked cancer registry data from England to assess the comparative effectiveness and cost-effectiveness of radical radiotherapy and radical cystectomy for OC-MIBC. A target trial emulation framework will be combined with advanced analytical methods to reduce the risk of bias and confounding.^25 27^ The target trial emulation framework applies RCT design principles to observational data by defining key study components, for example, eligibility criteria, treatment strategies and outcomes and can reduce the risk of biases commonly present in observational studies.^25^

The definitions of such key study components are informed by a previous RCT^10^ and observational studies comparing radical radiotherapy and radical cystectomy, pilot data from the NRDS Cancer Registry and input from an expert clinical panel. The clinical panel consisted of five consultant oncologists, six consultant urologists and two oncological nurses who met in June 2024 to provide their expert opinion on the study protocol. Input was sought on the eligibility criteria, treatment definitions, subgroups and effect size. The study will also include a CEA to assess the relative cost-effectiveness of definitive radiotherapy versus surgery for the overall eligible patient population with OC-MIBC and across subgroups.

### Data

Table 1 contains an overview of the datasets used in the study and their purpose. England’s NRDS Cancer Registry data will be used to identify individuals aged 18 and over diagnosed with OC-MIBC (T2-T4aN0M0) in 2015 to 2021 (inclusive).^4^ The Cancer Registry contains patient and tumour characteristics of all individuals diagnosed with malignant tumours in the UK and is collected in routine care delivery by NHS healthcare providers. The NRDS Cancer Registry contains information on vital status from the vital registry of the Office for National Statistics, that is, whether or not individuals have emigrated or been lost-to-follow-up before 31 December 2024. Further linkage will be made to the Hospital Episode Statistics (HES),^28^ which contain information on all inpatient stays and outpatient visits for NHS hospitals in UK. Inpatient stays will be used to identify patients who underwent a radical cystectomy. Inpatient admissions and outpatient records will be used to define the Hospital Frailty Risk Score^29^ and diagnoses with the individual comorbidities of the Charlson Comorbidity Index.^30^ NRDS observations will be linked to Systemic Anti-Cancer Therapy (SACT)^31^ data, which contain the type and dosage of drugs received for each cycle and regimen within NHS England. We will identify individuals who complete two to four cycles of a NAC regimen, as per national recommendations.^4 32^ The data will be linked to the National Radiotherapy Data Set (RTDS),^33^ which contains information on all NHS-funded radiotherapy regimens, to identify the receipt of radical radiotherapy.

**Table 1:** Overview of the linked datasets from the NRDS Cancer Registry and their purpose within the SORT-emulated target trial for OC-MIBC

Dataset	Purpose in the study
NDRS Cancer Registry	Identify individuals diagnosed with T2-T4aN0M0 urothelial OC-MIBC between 2015 and 2021 Collate patient and tumour characteristics, such as age at diagnosis, sex, tumour stage, histology and area-level income deprivation. Identify simultaneous upper tract transitional cancers and previous metastatic cancers.
SACT	Identify individuals who received 2–4 cycles of NAC. Collect data on the use of palliative and radical immuno-oncology and chemotherapy regimens.
HES inpatient and outpatient	Identify individuals who underwent radical cystectomy within 9 months of diagnosis and define time zero (date of cystectomy). Collect measured confounders, such as comorbidities and frailty. Identify women who were pregnant at diagnosis. Obtain resource utilisation, such as outpatient visits, additional surgical procedures and hospital admissions.
RTDS	Identify individuals who received radical radiotherapy within 9 months of diagnosis and define time zero (date of first dose of radical radiotherapy). Collect information on the further use of radical or palliative radiotherapy.

HES, Hospital Episodes Statistics; NAC, neoadjuvant chemotherapy; NRDS, National Disease Registration Service; OC-MIBC, organ-confined muscle-invasive bladder cancer; RTDS, radiotherapy data set; SACT, systemic anti-cancer therapy; SORT, surgery or radiotherapy.

### Emulated target trial design

We will use an emulated target trial framework^27^ to define the eligibility criteria, treatment strategies, and outcomes informed by a previous RCT^10^ comparing radical radiotherapy versus radical cystectomy (both following NAC) (table 2) to reduce the risk of confounding and other sources of bias. We will undertake advanced statistical analyses to reduce the risk of residual confounding (see planned statistical analyses for more detail).

**Table 2:** Overview of the emulated target trial elements and their conceptualisation for the SORT target trial for OC-MIBC

Protocol component	Emulation protocol
Inclusion criteria	Individuals aged ≥18 years with first primary histopathological confirmed urothelial OC-MIBC diagnosis (T2a-T4 N0 M0, ICD-10 code: C67, morphology codes: 8120 and 8130 (urothelial cancers) confirmed with histopathological testing) between 1 January 2015 and 31 December 2021 who received 2–4 cycles of neoadjuvant chemotherapy (Cis-Gem, Carbo-Gem, MVAC or AMVAC) followed by radical radiotherapy or radical cystectomy within 9 months of diagnosis
Exclusion criteria	Pregnancy at time of diagnosis
	Simultaneous upper tract transitional cancer
	Previous pelvic radiotherapy within 5 years prior to diagnosis Previous malignancy within 5 years prior to diagnosis
	Receipt of palliative radiotherapy or no treatment
Treatment strategies	Radical radiotherapy: External beam radiotherapy Radical surgery: Radical cystectomy
Assignment procedure	Randomisation will be emulated via double robust methods, such as TMLE or IPTW-RA for baseline covariates.
Time zero	Date of the start of definitive treatment (radical radiotherapy or radical cystectomy)
Follow-up	Follow-up begins on date of definitive treatment start (day 0) and ends 3 years after baseline
Outcome	Primary outcome: all-cause mortality at 3 years from the date of radiotherapy or cystectomy start
	Secondary outcomes: all-cause and bladder cancer-specific mortality at 1, 3 and 5 years; time to death; disease-free survival; incremental costs; and incremental cost-effectiveness (incremental net health benefits)
Causal contrast of interest	Intention-to-treat effect (analysing patients according to their allocated treatment, regardless of whether they did not fully adhere to treatment or switched to other treatments)

AMVAC, accelerated methotrexate, vinblastine, doxorubicin and cisplatin; Carbo-Gem, Carboplatin-Gemcitabine; Cis-Gem, Cisplatin-Gemcitabine; IPTW-RA, inverse probability weighting with regression adjustment; MVAC, methotrexate, vinblastine, doxorubicin and cisplatin; OC-MIBC, organ-confined muscle-invasive bladder cancer; SORT, surgery or radiotherapy; TMLE, targeted maximum likelihood estimation.

#### Eligibility criteria

**Figure 1:**
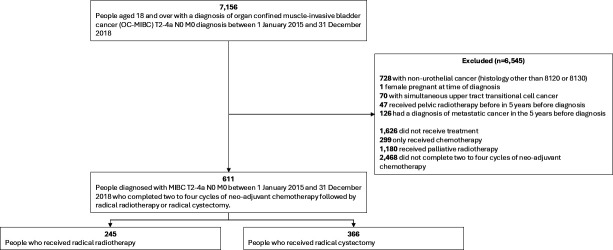
Flow diagram illustrating the identification of individuals who received neoadjuvant chemotherapy followed by radical cystectomy or radical radiotherapy for T2-4aN0M0 OC-MIBC diagnosed between 1 Jan 2015 and 31 Dec 2018. OC-MIBC, organ-confined muscle-invasive bladder cancer.

A flowchart of the eligible study participants based on NRDS-linked pilot data from 2015 to 2018 is presented in figure 1. The final study population for the base case analysis will include individuals aged ≥18 years with a histopathological confirmed diagnosis of urothelial OC-MIBC (T2-T4aN0M0) and who have completed two to four cycles of NAC followed by radical radiotherapy or radical cystectomy within 9 months of diagnosis. We will exclude individuals with a metastatic cancer diagnosis in the 5 years before their OC-MIBC diagnosis or individuals with a simultaneous diagnosis of upper-tract transitional cancer. We will exclude women who are pregnant at the time of OC-MIBC diagnosis, defined as a pregnancy-related admission or outpatient visit in HES up to 40 weeks after OC-MIBC diagnosis. Lastly, we will exclude individuals who previously received radiotherapy to the pelvic region.

#### Covariates and time zero

To address confounding and identify clinically relevant subgroups, we will obtain socio-demographic, clinical and patient characteristics from the NRD-linked data. From the NRDS Cancer Registry, we will obtain age at diagnosis, sex (female, male), ethnicity (White, Black, South-Asian and others), income deprivation quintile from the IMD, year of diagnosis, tumour stage (T2aN0M0, T3N0M0, T4N0M0) and tumour histology (urothelial carcinoma or transitional cell carcinoma). From SACT, we will collate the number of NAC cycles (two, three or four) and type of NAC regimen (Cis-Gem, Carbo-Gem, MVAC and AMVAC). History of diagnosis with relevant comorbidities from the Charlson Comorbidity Index,^30^ for example, myocardial infarction (MI), heart failure (HF), peripheral vascular disease (PVD) and diabetes, and Hospital Risk Frailty Score^29^ will be collected from HES using ICD-10 codes of secondary diagnoses. We will also estimate the number of hospitalisations in the 5 years before OC-MIBC diagnosis.

To conduct an emulated target trial, a time zero needs to be defined. Time zero is equivalent to the time of randomisation in an RCT when all the eligibility criteria are met, the treatment strategies start and follow-up begins. We define the start of definite treatment, that is, the day that the first dose of radical radiotherapy is administered, or the day that the patient has radical cystectomy, as time zero. Covariates will also be included to address seasonality, that is, the month of treatment and waiting time, that is, the time between diagnosis and day 0. In addition, we will also include NHS regions to account for regional variation.

#### Treatment strategies

Table 3 contains an overview of the NAC radiotherapy regimens and surgical definitions included. The definitions are based on guidelines from NICE,^4^ the Royal College of Radiologists,^34^ published literature^35 36^ and the clinical panel. We defined a ‘treatment window’ of 30 days before and 9 months after OC-MIBC diagnosis to accommodate the prioritisation of a pathology diagnosis as recommended by the European Network of Cancer Registry^37^ and to allow for variation across NHS trusts and over time in the time patients with similar prognoses may wait for either radical treatment, including the time required to have two to four cycles of NAC.

**Table 3:** Overview of the NAC and radical radiotherapy regimens and definition for radical cystectomy

NAC regimen	Reference
Cisplatin–gemcitabine	
21-day cycle with cisplatin on day 1 and gemcitabine on days 1 and 8	Royal College of Radiologists—Bladder cancer consensus statement^34^
28-day cycle with cisplatin on day 1 and gemcitabine on days 1, 8 and 15	
21-day cycle with cisplatin on days 1 and 8 and gemcitabine on days 1 and 8	
MVAC	
28-day cycle with methotrexate on days 1, 15, and 22; vinblastine on days 2, 15 and 22 and cisplatin and doxorubicin hydrochloride on day 2	Royal College of Radiologists—Bladder cancer consensus statement^34^
Methotrexate, cisplatin and vinblastine (AMVAC)	
14-day cycle with methotrexate, cisplatin, doxorubicin and vinblastine on day 1	International collaboration of trialists on behalf of the Medical Research Council Advanced Bladder Cancer Working Party^35^
14-day cycle with methotrexate, vinblastine, doxorubicin, and cisplatin on days 1 and 7	
Carboplatin–gemcitabine	
21-day cycle with carboplatin on day 1 and gemcitabine on days 1 and 8	NICE, Einerhand *et al* 2022^4 60^
28-day cycle with carboplatin on day 1 and gemcitabine on days 1, 8 and 15	
21-day cycle with carboplatin on day 1 and day 8, and gemcitabine on days 1 and 8	
AMVAC	
14-day cycle with methotrexate, cisplatin, doxorubicin and vinblastine on day 1	Blick *et al* 2012^36^
14-day cycle with methotrexate, vinblastine, doxorubicin and cisplatin on days 1 and 7	
Radical radiotherapy regimens	
52.5-55 Gy in 20 fractions	Royal College of Radiologists^34^
60-64 Gy in 30–32 fractions	
Radical cystectomy	
OPCS-4: M34.1 cystprostatectomy	Input from clinical panel
OPCS-4: M34.2 cystourethrectomy	
OPCS-4: M34.3 cystectomy NEC	
OPSC-4: M34.8 other specified total excision of bladder	
OPCS-4: M34.9 unspecified total excision of bladder	

AMVAC, accelerated methotrexate, vinblastine, doxorubicin, cisplatin; MVAC, methotrexate, vinblastine, doxorubicin, cisplatin; NAC, neoadjuvant chemotherapy; NICE, National Institute for Health Care and Excellence.

#### Follow-up period and outcomes

The primary outcome is 3-year all-cause mortality from the start of treatment (time zero). Secondary outcomes include all-cause mortality at 1 and 5 years and bladder-cancer-specific mortality at 1, 3 and 5 years. We will calculate bladder-cancer mortality using a cause-of-death approach and will consider all deaths that include bladder cancer (ICD-10: C67) as a primary or secondary cause of death as attributable to bladder cancer. We will fit multivariable time-to-event models^38 39^ to compare the effect of radical radiotherapy versus radical cystectomy on time to death. We will include readmissions at 90 days as a proxy for adverse outcomes related to treatment. We will use information on reasons and routes of admission (eg, emergency department) to identify admissions attributable to grade 3 or 4 adverse outcomes following either treatment modality. Lastly, we will identify any recurrence (loco-regional or distant) by adapting an approach developed for bowel cancer to estimate disease-free survival.^40^

Data on the 3-year all-cause mortality (primary outcome) was complete for all eligible individuals in the NRDS pilot data. Small numbers of missings were observed for some baseline covariates, that is, missing ethnicity <3%. In the main analysis, we will undertake a complete case analysis (see alternative analysis).

#### Causal contrast of interest

The analysis will take an intention-to-treat (treatment policy) approach whereby all individuals are assigned and will contribute to their treatment arm based on whether they had definitive radiotherapy or surgery irrespective of the subsequent treatment received. Individuals who received salvage cystectomy or radical radiotherapy following their initial treatment will thus continue to contribute to the treatment arm to which they were assigned. Individuals will remain exposed until death or the end of the follow-up period, the latest being 31 December 2024.

#### Sample size calculations

An absolute risk difference of 8.5% points between the two comparison groups in all-cause mortality at 3 years was defined as clinically relevant based on a previous RCT^10^ and expert opinion provided by the clinical panel. We conducted a sample size calculation using the NRDS pilot data from 2015 to 2018 to estimate the 3-year all-cause mortality (24%) for patients who received radical cystectomy. In the NRDS pilot data, 42% receive radiotherapy and 58% receive cystectomy. Table 4 presents the sample sizes that can detect different effect sizes with 80% power at a 5% level of statistical significance (two-sided). Based on the NRDS pilot data between 2015 and 2018, we expect a final sample size of 1200 patients.

**Table 4:** Required sample size (N) to estimate the comparative effectiveness of radical radiotherapy compared with radical cystectomy, both following NAC, for OC-MIBC. Assuming 80% power, 5% level of statistical significance and an 8.5% margin

Effect size	Radical cystectomy	Radical radiotherapy	Total
0.0%	372	269	641
2.5%	228	165	393
5.0%	155	112	268
8.5%	101	73	174

NAC, neoadjuvant chemotherapy; OC-MIBC, organ-confined muscle-invasive bladder cancer.

### Cost-effectiveness analysis

#### Overview

The comparative cost-effectiveness of radical radiotherapy versus radical cystectomy, both after NAC for OC-MIBC, will be assessed within the emulated target trial framework using a multistate modelling approach.^41^ The CEA will take an NHS perspective and report costs, outcomes, and cost-effectiveness over a lifetime horizon. A lifetime horizon is consistent with NICE guidance, which recommends adopting a sufficiently long horizon to capture important differences in costs and health outcomes between treatment alternatives.^42^ The model will describe the clinical and cost consequences resulting from different treatment strategies through the estimation of recurrence and death transition probabilities using survival regression models with standard parametric distributions (eg, exponential, Weibull, lognormal, Gompertz).^41^ We will include individual-level resource utilisation and mortality data from the NRDS Cancer Registry and linked datasets. Unit costs and health-related quality of life (HRQoL) estimates will be extracted from the costing database and published literature.^10 23 43^ We will conduct a double robust analysis to account for observed confounding (see Planned statistical analyses).

#### Resource utilisation and unit costs

We will use the NRDS-linked data to obtain information on resource use that is expected to drive incremental costs, including the delivery of radical radiotherapy, radical cystectomy, hospitalisations for readmissions, palliative care, outpatient visits and subsequent treatments, such as subsequent surgeries, systemic therapies and radiotherapy with radical or palliative intent.

We will extract the relevant unit costs from the NHS Cost Collection^44^ and the Personal Social Services Unit (PSSRU)^45^ cost databases and combine these with resource use data to estimate the cost of surgical procedures, radical radiotherapy and service delivery. The individual costs will be summed to calculate total costs over the relevant disease stages required to populate the lifetime cost-effectiveness model.

#### Measures in the CEA

From the NRDS linked to ONS data, the time to death will be available for patients up to the maximum period of follow-up. Cancer recurrence will be obtained from NRDS data using diagnoses and procedures captured in hospital admission, diagnosis with new tumours and subsequent treatment receipt (eg, surgery, systemic therapy and radiotherapy) using an approach developed for bowel cancer, which we will apply for OC-MIBC.^40^ We will compare the adjusted proportions of cancer recurrences, common severe events and disease progression after radical radiotherapy versus radical cystectomy.

The cost-effectiveness model will be populated with HRQoL estimates from the literature^46^,^50^ for the respective disease stages (eg, before vs after cancer recurrence). The outcomes for the CEA will be presented using appropriate CEA metrics, such as the incremental net benefit by valuing the incremental QALYs with alternative thresholds for the cost per QALY gain, including those specified by NICE (eg, £25,000 and £35,000).

### Planned statistical analyses

#### Base case (main) analysis

We will apply advanced statistical methods that aim to reduce the risk of confounding that is due to differences in observed baseline characteristics between the comparison groups. Specifically, we will use a double robust analysis, such as targeted maximum likelihood estimation (TMLE)^51^ or inverse probability treatment weight with regression adjustment (IPTW-RA),^52^ to estimate the comparative effectiveness and cost-effectiveness of radical radiotherapy versus radical cystectomy both after NAC for OC-MIBC and account for observed confounding. Double robust methods involve fitting a propensity score model, which estimates the probability of receiving treatment accounting for observed characteristics. The propensity score is then incorporated in a second model with the received treatment and covariates to estimate the treatment effect. Double robust methods provide some protection against model specification, as they can yield unbiased treatment effects as long as at least one of the models is correctly specified (known as the double robust property).^53^

Four assumptions need to be met to estimate unbiased treatment effects using double robust methods. First, it assumes that all confounding has been observed and is accounted for in the model. Second, all patients have a non-zero probability of receiving the alternative treatment after accounting for observed confounding. Third, the observed outcomes correspond to the observed treatments (consistency), which implies that the treatment received and the resultant outcomes can both be identified within the data. Fourth, the outcomes of one individual are not dependent on the treatment of other individuals in the study population.^54^

We will use a double-robust method to estimate the comparative effectiveness and cost-effectiveness of radical radiotherapy versus radical cystectomy after neo-adjuvant chemotherapy receipt for OC-MIBC and pre-specified subgroups, including sex, age, performance status, Charlson comorbidity index and hospital frailty risk score.^29^

#### Alternative analyses

Alternative analyses will be conducted that fall broadly under four different categories to test the robustness of the base case result. The first group will assess the robustness of the results against the eligibility criteria. For example, we will exclude patients that did not receive either definite treatment within 6 months (vs 9 months) of diagnosis. We will also consider excluding patients who receive carboplatin—gemcitabine as neo-adjuvant chemotherapy. Second, we will consider the possibility that after NAC, cancer stage may be misclassified for some people who have definitive radiotherapy and the related problem that for a subsample of patients, some covariate information will be missing. We will consider alternative analyses that make alternative assumptions (eg, multiple imputation). Third, we will also consider the impact of COVID-19 by conducting an analysis that excludes patients that were treated during the first wave of COVID-19 (1 February to 30 June 2020). Fourthly, we will use quantitative bias analysis methods, such as E-value means, to scrutinise the plausibility of assuming that all confounding is observed and that it has been accounted for by the proposed double robust methods.^55 56^ Fifth, the CEA analysis will consider alternative sources for HRQoL and conduct sensitivity analysis to assess the impact of different assumptions on the treatment effects for various outcomes.

### Strengths and limitations

This study will assess the comparative effectiveness and cost-effectiveness of radical radiotherapy versus radical cystectomy after NAC for OC-MIBC. The use of England’s national linked cancer registry ensures a large and representative sample that can inform clinical decision-making. Furthermore, this allows us to report comparative effectiveness for clinically relevant subgroups that would normally be underrepresented in RCTs. The study protocol bears strong resemblance to the RCT^10^ we intend to emulate but also allows for the full representation of subgroups that would be underrepresented in RCTs. The expert clinical panel has informed key aspects of the study, including the eligibility criteria, treatment definitions, outcome measures and anticipated effect sizes, to enhance the validity and clinical relevance.

A limitation of the study is that the NRDS Cancer Registry only captures stage at diagnosis and not after NAC. We thus have limited information on the tumour stage at definitive treatment start, particularly for the radiotherapy patients. To accommodate for potential differences in tumour stage at treatment start, we will consider adjusting the analyses for the type and number of cycles of NAC received and the time between diagnosis and treatment start. We will also consider alternative analyses that make contrasting assumptions. The NRDS-linked data does not capture all outcomes of interest, such as adverse events, HRQoL and cancer recurrence. The concurrent use of radiosensitisers is not considered in the treatment definition since the RTDS dataset also does not capture the concurrent use of radiosensitisers, which can result in improved locoregional control compared with radical radiotherapy alone, according to RCTs.^57 58^

While the combination of the emulated target trial design with double robust methods reduces the risk of confounding, the statistical approach suggested in this protocol assumes no unmeasured confounding to identify treatment effects. Non-randomised studies are typically subject to unmeasured confounding, which, if not addressed, may lead to biased treatment effect estimates and misleading cost-effectiveness results. Methods that explicitly account for unmeasured confounding, such as instrumental variables, require a larger sample size than the one anticipated in this study.^59^ In this case, the use of quantitative bias analysis methods can be helpful in assessing the plausibility of the *no unmeasured confounding* assumption by quantifying the effect of unmeasured confounding on the results.^56^

### Patient and public involvement

Two public and patient involvement (PPI) representatives with lived experience of cancer contributed to the study conceptualisation throughout the study, prior to securing funding and during the protocol development. The PPI representatives highlighted the relevance of capturing individual characteristics, including age, fitness and tumour stage, in the study design. A PPI panel consisting of eight individuals from diverse backgrounds with lived experience of cancer as a patient, carer or community support worker was convened by the PPI representatives and the PPI study lead. The PPI panel meets regularly and contributes to key elements of the study design, including outcomes and subgroups. The PPI representatives and panel members will continue to assist in identifying key messages and ensuring that any communication about the study results is accessible to patients and the general public.

Furthermore, three clinical experts, a consultant clinical oncologist (AC) and a consultant urologist (JC), and a specialist clinical oncology trainee (HB) are integral to the study team. Their expertise contributed to defining the eligibility criteria, treatment definitions, planned analyses and outcome measures.

### Deviations from protocol

Potential deviations from the published protocol will be published on the study website https://www.lshtm.ac.uk/research/centres-projects-groups/sort.

## Ethics and dissemination

### Ethics

This study will use data from the national cancer registry in which data are provided by patients and collected by the NHS as part of routine care (Data sharing agreement: DARS-NIC-656757-J8V9D-v2.3). Patients can opt out of data collection in the cancer registry data. Since the collected data do not contain identifiable information, individual consent was not required. Independent ethics approval was obtained by the London School of Hygiene and Tropical Medicine Research Ethics Committee (reference number 29 717–1). The proposed analysis and future interpretation of the results are the responsibility of the authors.

### Dissemination

We will maintain ongoing collaboration with our expert clinical colleagues and PPI representatives throughout the study to share clinical and health economic outputs and ensure the findings are translated into clinical recommendations for patients with OC-MIBC. We aim to publish the results in open-access journals and present the findings at scientific and clinical conferences. Methodological developments and subsequent research findings will be disseminated to support the application of observational evidence in improving healthcare delivery and optimising resource allocation.

## References

[1] Bladder cancer statistics (2015). Cancer Research UK.

[2] Exarchakou A, Rachet B, Belot A (2018). Impact of national cancer policies on cancer survival trends and socioeconomic inequalities in England, 1996-2013: population based study. BMJ.

[3] Boustead GB, Fowler S, Swamy R (2014). Stage, grade and pathological characteristics of bladder cancer in the UK: British Association of Urological Surgeons (BAUS) urological tumour registry. BJU Int.

[4] NICE (2015). Bladder cancer: diagnosis and management. https://www.nice.org.uk/guidance/ng2/resources/bladder-cancer-diagnosis-and-management-pdf-51036766405.

[5] John JB, Varughese MA, Cooper N (2021). Treatment Allocation and Survival in Patients Diagnosed with Nonmetastatic Muscle-invasive Bladder Cancer: An Analysis of a National Patient Cohort in England. Eur Urol Focus.

[6] Varughese M, Treece S, Drinkwater KJ (2019). Radiotherapy Management of Muscle Invasive Bladder Cancer: Evaluation of a National Cohort. Clin Oncol.

[7] GIRFT (2022). Urology: towards better care for patients with bladder cancer. https://www.gettingitrightfirsttime.co.uk/wp-content/uploads/2021/12/Urology_2021-12-10_Guidance_Bladder-cancer.pdf.

[8] Bladder: Malignant - Treatment Breakdown by Alliance, 2013-2021.

[9] Barki C, Rahmouni HB, Labidi S (2021). The Impact of Socioeconomic Variables Status on Bladder Cancer Treatment Outcomes during the COVID-19 Pandemic. *OAlib*.

[10] Huddart RA, Birtle A, Maynard L (2017). Clinical and patient-reported outcomes of SPARE - a randomised feasibility study of selective bladder preservation versus radical cystectomy. BJU Int.

[11] Munro NP, Sundaram SK, Weston PMT (2010). A 10-Year Retrospective Review of a Nonrandomized Cohort of 458 Patients Undergoing Radical Radiotherapy or Cystectomy in Yorkshire, UK. Int J Radiat Oncol.

[12] Kotwal S, Choudhury A, Johnston C (2008). Similar Treatment Outcomes for Radical Cystectomy and Radical Radiotherapy in Invasive Bladder Cancer Treated at a United Kingdom Specialist Treatment Center. Int J Radiat Oncol.

[13] Yamamoto Y, Kawashima A, Morishima T (2023). Comparative Effectiveness of Radiation Versus Radical Cystectomy for Localized Muscle-Invasive Bladder Cancer. Adv Radiat Oncol.

[14] Zlotta AR, Ballas LK, Niemierko A (2023). Radical cystectomy versus trimodality therapy for muscle-invasive bladder cancer: a multi-institutional propensity score matched and weighted analysis. Lancet Oncol.

[15] Zhou YX, Hu QC, Zhu YJ (2023). Comparison of trimodality therapy and neoadjuvant chemotherapy combined with radical cystectomy for the survival of muscle-invasive bladder cancer: a population-based analysis. Eur J Med Res.

[16] Su X, Dong C, Liao W (2023). Oncological effectiveness of bladder-preserving trimodal therapy versus radical cystectomy for the treatment of muscle-invasive bladder cancer: a system review and meta-analysis. World J Surg Onc.

[17] Fahmy O, Khairul-Asri MG, Schubert T (2018). A systematic review and meta-analysis on the oncological long-term outcomes after trimodality therapy and radical cystectomy with or without neoadjuvant chemotherapy for muscle-invasive bladder cancer. Urol Oncol Semin Orig Investig.

[18] Vashistha V, Wang H, Mazzone A (2017). Radical Cystectomy Compared to Combined Modality Treatment for Muscle-Invasive Bladder Cancer: A Systematic Review and Meta-Analysis. Int J Radiat Oncol.

[19] Williams SB, Shan Y, Ray-Zack MD (2019). Comparison of Costs of Radical Cystectomy vs Trimodal Therapy for Patients With Localized Muscle-Invasive Bladder Cancer. JAMA Surg.

[20] Golla V, Shan Y, Farran EJ (2022). Long term cost comparisons of radical cystectomy versus trimodal therapy for muscle-invasive bladder cancer. Urol Oncol Semin Orig Investig.

[21] Kool R, Yanev I, Hijal T (2022). Trimodal therapy vs. radical cystectomy for muscle-invasive bladder cancer: A Canadian cost-effectiveness analysis. Can Urol Assoc J.

[22] Suskovic N, Raldow AC, Royce TJ (2020). Cost-effectiveness of radical cystectomy versus trimodality therapy for muscle invasive bladder cancer. JCO.

[23] Joyce DD, Wymer KM, Graves JA (2025). Cost-Effectiveness of Trimodal Therapy and Radical Cystectomy for Muscle-Invasive Bladder Cancer. *JAMA Netw Open*.

[24] Smith AB, McCabe S, Deal AM (2021). Quality of Life and Health State Utilities in Bladder Cancer. Bladder Cancer.

[25] Hernán MA, Robins JM (2016). Using Big Data to Emulate a Target Trial When a Randomized Trial Is Not Available. Am J Epidemiol.

[26] NCRAS National Health System Digital.

[27] Hernán MA, Wang W, Leaf DE (2022). Target Trial Emulation: A Framework for Causal Inference From Observational Data. JAMA.

[28] Herbert A, Wijlaars L, Zylbersztejn A (2017). Data Resource Profile: Hospital Episode Statistics Admitted Patient Care (HES APC). Int J Epidemiol.

[29] Gilbert T, Neuburger J, Kraindler J (2018). Development and validation of a Hospital Frailty Risk Score focusing on older people in acute care settings using electronic hospital records: an observational study. The Lancet.

[30] Charlson Comorbidity Index (CCI).

[31] Bright CJ, Lawton S, Benson S (2020). Data Resource Profile: The Systemic Anti-Cancer Therapy (SACT) dataset. Int J Epidemiol.

[32] Royal College of Radiologists Bladder Cancer - RCR Consensus Statements.

[33] Sandhu S, Sharpe M, Findlay Ú (2023). Cohort profile: radiotherapy dataset (RTDS) in England. BMJ Open.

[34] Royal College of Radiologists (2023). Bladder cancer - rcr consensus statements. https://www.rcr.ac.uk/media/00nfoiko/rcr-publications_bladder-cancer-rcr-consensus-statements_may-2023.pdf.

[35] International collaboration of trialist on behalf of the Medical Research Council Advanced Bladder Cancer Working Party (1999). Neoadjuvant cisplatin,methotrexate,and vinblastine chemotherapy for muscle-invasive bladder cancer: a randomised controlled trial. The Lancet.

[36] Blick C, Hall P, Pwint T (2012). Accelerated methotrexate, vinblastine, doxorubicin, and cisplatin (AMVAC) as neoadjuvant chemotherapy for patients with muscle-invasive transitional cell carcinoma of the bladder. Cancer.

[37] Tyczynski J, Démaret E, Parkin D Standards and Guidelines for Cancer Registration in Europe.

[38] Royston P, Parmar MKB (2002). Flexible parametric proportional-hazards and proportional-odds models for censored survival data, with application to prognostic modelling and estimation of treatment effects. Stat Med.

[39] Pohar Perme M, Estève J, Rachet B (2016). Analysing population-based cancer survival - settling the controversies. BMC Cancer.

[40] NIHR Funding and Awards Identifying Cancer Recurrence within Patient Care Pathways across Linked National Clinical Datasets.

[41] Williams C, Lewsey JD, Mackay DF (2017). Estimation of Survival Probabilities for Use in Cost-effectiveness Analyses: A Comparison of a Multi-state Modeling Survival Analysis Approach with Partitioned Survival and Markov Decision-Analytic Modeling. Med Decis Making.

[42] NICE (2025). NICE Technology Appraisal and Highly Specialised Technologies Guidance: The Manual.

[43] Joyce DD, Sharma V, Williams SB (2023). Cost-Effectiveness and Economic Impact of Bladder Cancer Management: An Updated Review of the Literature. Pharmacoeconomics.

[44] NHS (2021). National cost collection 2019/20. https://www.england.nhs.uk/costing-in-the-nhs/national-cost-collection/#ncc1819.

[45] Jones B (2021). Costs of health and social care 2021. https://www.pssru.ac.uk/project-pages/unit-costs/unit-costs-of-health-and-social-care-2021/.

[46] Chaballout BH, Chang EM, Parikh NR (2023). Assessing utilities for muscle-invasive bladder cancer-related health states. Urol Oncol Semin Orig Investig.

[47] Mason SJ, Downing A, Wright P (2018). Health-related quality of life after treatment for bladder cancer in England. Br J Cancer.

[48] Rogers Z, Glaser A, Catto JWF (2024). Health-related quality of life after a diagnosis of bladder cancer: a longitudinal survey over the first year. BJU Int.

[49] Catto JWF, Downing A, Mason S (2021). Quality of Life After Bladder Cancer: A Cross-sectional Survey of Patient-reported Outcomes. Eur Urol.

[50] Chang EM, Parikh NR, Min Y (2020). Assessing Utilities for Muscle-Invasive Bladder Cancer-Related Health States. Int J Radiat Oncol.

[51] van der Laan MJ, Rubin D (2006). Targeted Maximum Likelihood Learning. Int J Biostat.

[52] Rosenbaum PR (1987). Model-Based Direct Adjustment. J Am Stat Assoc.

[53] Bang H, Robins JM (2005). Doubly robust estimation in missing data and causal inference models. Biometrics.

[54] Angrist J, Imbens G (1995). Identification and Estimation of Local Average Treatment Effects. National Bureau of Economic Research.

[55] Leahy TP, Duffield S, Kent S (2022). Application of quantitative bias analysis for unmeasured confounding in cost–effectiveness modelling. J Comp Eff Res.

[56] Leahy TP, Kent S, Sammon C (2022). Unmeasured confounding in nonrandomized studies: quantitative bias analysis in health technology assessment. J Comp Eff Res.

[57] James ND, Hussain SA, Hall E (2012). Radiotherapy with or without Chemotherapy in Muscle-Invasive Bladder Cancer. N Engl J Med.

[58] Hoskin PJ, Rojas AM, Saunders MI (2009). Carbogen and nicotinamide in locally advanced bladder cancer: Early results of a phase-III randomized trial. Radiother Oncol.

[59] Boef AGC, Dekkers OM, Vandenbroucke JP (2014). Sample size importantly limits the usefulness of instrumental variable methods, depending on instrument strength and level of confounding. J Clin Epidemiol.

[60] Einerhand SMH, Black AJ, Zargar H (2022). Multicenter evaluation of neoadjuvant and induction gemcitabine–carboplatin versus gemcitabine–cisplatin followed by radical cystectomy for muscle-invasive bladder cancer. World J Urol.

