# Supplementary material for: Surgery or radiotherapy for early-stage cancer study protocol for an emulated target trial of radical radiotherapy versus radical cystectomy, with either following neoadjuvant chemotherapy, for organ-confined muscle-invasive bladder cancer
Source: BMJ Open. 2026 Jun 11;16(6):e119596. doi: 10.1136/bmjopen-2026-119596 (PMC13264877; doi:10.1136/bmjopen-2026-119596)
Supplement: Supplementary file 1 [file bmjopen-2026-119596.reviewer_comments.pdf]

PEER REVIEW HISTORY

BMJ Open publishes all reviews undertaken for accepted manuscripts. Reviewers are asked to complete a checklist review form and are provided with free text boxes to elaborate on their assessment. These free text comments are reproduced below.

ARTICLE DETAILS

Title (Provisional)

Surgery Or RadioTherapy for early-stage cancer study (SORT) protocol for an Emulated Target Trial of radical radiotherapy versus radical cystectomy, with either following neoadjuvant chemotherapy, for organ-confined muscle-invasive bladder cancer (OC-MIBC).

Authors

Kagenaar, Eva; Lugo-Palacios, David Gibran; Bromley, Hannah; Aggarwal, Ajay; Hutchings, Andrew; O’Neill, Stephen; Rachet, Bernard; Cresswell, Jo; Choudhury, Ananya; Grieve, Richard

---

This article was not externally reviewed at BMJ Open. Protocols that have been independently assessed prior to submission to BMJ Open are usually fast-tracked to publication on the grounds that further substantial changes will not be possible. This independent assessment will usually be external, independent review for both a substantial grant award from a non-commercial or government funder and ethics approval.
